# Skeletal effects following developmental flame-retardant exposure are specific to sex and chemical class in the adult Wistar rat

**DOI:** 10.3389/ftox.2023.1216388

**Published:** 2023-07-27

**Authors:** Stacy Schkoda, Brian Horman, Shannah K. Witchey, Anton Jansson, Soraia Macari, Heather B. Patisaul

**Affiliations:** ^1^ Department of Biological Sciences, North Carolina State University, Raleigh, NC, United States; ^2^ National Toxicology Program, National Institute of Environmental Health Sciences, Research Triangle Park, NC, United States; ^3^ Analytical Instrumentation Facility, North Carolina State University, Raleigh, NC, United States; ^4^ Department of Restorative Dentistry, Federal University of Minas Gerais, Belo Horizonte, Minas Gerais, Brazil; ^5^ Center for Human Health and the Environment, North Carolina State University, Raleigh, NC, United States

**Keywords:** endocrine disrupting chemicals, flame retardants, sex difference, osteotoxicology, computed tomography, bone

## Abstract

**Introduction:** Accumulating evidence reveals that endocrine disrupting chemicals (EDCs) can disrupt aspects of metabolic programming, suggesting that skeletal development may be at risk, a possibility that is rarely examined. The commercial flame retardant (FR) mixture, Firemaster 550 (FM 550), has repeatedly been shown to negatively influence metabolic programming, raising concerns that skeletal integrity may consequently be impaired. We have previously shown that gestational and lactational exposure to 1,000 µg FM 550 negatively affected sex-specific skeletal traits in male, but not female, rats assessed at 6 months of age. Whether this outcome is primarily driven by the brominated (BFR) or organophosphate ester (OPFR) portions of the mixture or the effects persist to older ages is unknown.

**Materials and methods:** To address this, in the present study, dams were orally exposed throughout gestation and lactation to either 1,000 μg BFR, 1,000 µg OPFR, or 2,000 µg FM 550. Offspring (*n* = 8/sex/exposure) were weaned at PND 21 and assessed for femoral cortical and trabecular bone parameters at 8 months of age by high-resolution X-ray micro-computed tomography (micro-CT). Serum levels of serotonin, osteocalcin, alkaline phosphatase, and calcium were quantified.

**Results:** FM 550 affected both sexes, but the females were more appreciably impacted by the OPFRs, while the males were more vulnerable to the BFRs.

**Conclusion:** Although sex specificity was expected due to the sexual dimorphic nature of skeletal physiology, the mechanisms accounting for the male- and female-specific phenotypes remain to be determined. Future work aims to clarify these unresolved issues.

## Introduction

The developmental origins of health and disease (DOHaD) hypothesis broadly considers how environmental influences incurred during important early developmental stages impart lasting deficits throughout life (Haugen et al., 2015). These deficits may include metabolic, neurocognitive, or structural effects including skeletal effects. The impact of the environment is not inconsequential as, according to the World Health Organization, almost one quarter of global deaths are linked to environmental factors. One subset of consequential environmental factors includes endocrine disrupting chemicals (EDCs), defined by the Endocrine Society as an exogenous chemical, or a mixture of chemicals, that interferes with any aspect of hormone action (Gore et al., 2015). Although, historically, the vast majority of EDC research has focused on reproductive and neural targets, emerging work suggests that metabolic reprogramming is also a primary consequence of developmental EDC exposure (Janesick and Blumberg, 2011; Schneider et al., 2014; Heindel et al., 2017; Sargis and Simmons, 2019). It has been hypothesized that, based on *in vitro* work, some EDCs can promote adiposity at the expense of osteoblast production from mesenchymal stem cells (Heindel et al., 2022). We have shown that developmental exposure to the commercial flame-retardant (FR) mixture Firemaster 550 (FM 550) sex-specifically compromises bone composition in Wistar rats (Macari et al., 2020). Here, we followed up on that aforementioned work to determine which chemical class in the commercial mixture may be driving the phenotype.

FRs are used in a myriad of household products such as carpets, baby and children’s products, electronics, building materials, and polyurethane foam used in gyms, furniture, and mattresses. As such, exposure is widespread and higher in children than in adults (Stapleton et al., 2008; Stapleton et al., 2012; Carignan et al., 2013). The use of FM 550 has changed dramatically in less than two decades. It was the second most detected FR in furniture products in the United States in the early 2000s, but its use appears to have decreased in favor of contemporary mixtures containing either a subset of FM 550 components with other FRs or the same components at different concentrations (Stapleton et al., 2008; Stapleton et al., 2009; Stapleton et al., 2012; Phillips et al., 2017). Thus, understanding the potential toxicity of the component classes is critically important. FM 550 is a roughly 50:50 mixture of brominated (BFR) and organophosphate ester (OPE) flame-retardant (OPFR) chemicals, some of which are also used in other applications, including personal care products (Mendelsohn et al., 2016; Phillips et al., 2017). In cellular and animal models, FM 550 has been characterized as obesogenic and endocrine-disrupting (Krumm et al., 2018; Heindel et al., 2022), with our group and others showing that developmental FM 550 exposure sex-specifically impairs neurodevelopment, socioemotional behaviors, and, possibly, skeletal development (Patisaul et al., 2013a; Belcher et al., 2014; Gillera et al., 2019; Macari et al., 2020; Witchey et al., 2020).

*In vitro*, FM 550 has been shown to interact with PPARγ to promote adipogenesis in mesenchymal stem cells at the expense of osteogenesis, even when cultured under pro-osteogenic conditions. Upon differentiating 3T3-L1 cells in the presence of triphenyl phosphate (TPHP), an OFPR found in the FM 550 mixture, lipid size and volume were increased and the expression of multiple pro-adipogenic genes was upregulated (Tung et al., 2017a; Tung et al., 2017b; Cano-Sancho et al., 2017; Stapleton et al., 2017). This suggests that FM 550 could shift metabolic programming toward adipose development at the expense of bones. Given the widespread use of FM 550 components and their known adverse effects during development on multiple systems, it is important to further understand their potential effects on skeletal health.

In fish models, abnormal skeletal development, gene network dysregulation, and gross morphological impairment of skeletal organization has been observed in response to developmental BFR or OPFR exposure (Macaulay et al., 2017; Alzualde et al., 2018). Aquatic species, such as medaka and zebrafish, are often used for high-throughput screening and are accepted models of skeletal development due to conserved pathways with human development (Padilla et al., 2009; Shanthanagouda et al., 2014; Witten et al., 2017; Bergen et al., 2019; Lleras-Forero et al., 2020). In medaka, developmental exposure to two OPFRs present in FM 550 induced abnormal pectoral fin development, spinal curvature, and dysregulation of BMP signaling networks critical for early cartilage and bone development (Hong et al., 2021). In zebrafish, major signaling pathways such as MAPK were also disrupted following OPFR exposure (Kling and Förlin, 2009). Both species also displayed sex-specific disruption of the hypothalamic–pituitary–gonadal (HPG) axis (Liu et al., 2013; Saunders et al., 2015; Zhu et al., 2018), as well as behavioral disruptions akin to those observed in mammalian models, demonstrating species concordance (Glazer et al., 2018; Gillera et al., 2019). Evidence of sex specificity has also been reported. For example, in response to exposure to an OPFR mixture, adult male zebrafish had increased corticotropin-releasing hormone (*crh*) and thyroid-stimulating hormone (*tsh*) transcription in the brain and downregulation of thyroglobulin and deiodinase 2 in the thyroid and liver, while females displayed downregulated *crh* and *tsh* (Wang et al., 2013; Kim et al., 2015; Liu et al., 2019).

In rodent models, gestational and lactational exposure to FR mixtures negatively affects neurodevelopment, liver and thyroid function, global metabolism, and skeletal development (Patisaul et al., 2013a; Phillips et al., 2016; Adams et al., 2020; Macari et al., 2020). Sprague–Dawley rat pups exposed to an environmentally relevant BFR-based mixture throughout gestation and lactation failed to completely ossify vertebrae and phalanges by PND 4 (Berger et al., 2014). In an *ex vivo* murine limb bud model, exposure to OPFRs or BFRs severely stunted the organization of COL10A1, an early marker of chondrogenesis in mammals that is critical to proper skeletal growth, and antagonized Hedgehog signaling (Yan and Hales, 2019; Yan and Hales, 2020). It is unclear if or how these deficits persist into adulthood.

Previously, our group showed in a pilot study that the gross bone mineral density and microarchitecture in cortical and trabecular structures was disrupted in male (but not female) Wistar rats that were gestationally and lactationally exposed to a single dose of FM 550 (1,000 μg/kg bw; ∼3.3 mg/kg bw to the dam) (Macari et al., 2020). FM 550-exposed males had altered histomorphology including a decreased number of osteoblasts and increased proportion of yellow marrow (Macari et al., 2020). No changes in osteoclasts were observed in either sex. While this aforementioned study established FM 550 as an environmental contaminant that can sex-specifically impair skeletal outcomes, it is unclear which chemical class in the mixture is primarily responsible for the effects or if it is a unique effect of the full mixture. To begin to address this, here, we assessed the effects of each FR class and the full mixture on cortical and trabecular bone and morphology of the femur in Wistar rats. The approach utilized advanced X-ray computed tomography instrumentation which was unavailable for our prior related study; thus, we included a subset of those animals in the present study to validate our updated approach (Macari et al., 2020).

The identification of serum biomarkers for compromised skeletal integrity would be useful for future studies probing the mechanism of action and potential human risk. Consequently, we examined circulating serotonin (5-HT) and osteocalcin (OCN) levels. Our group has previously shown that developmental FM 550 exposure alters serotonergic (5-HTergic) innervation of the rat fetal forebrain, disrupts placental tryptophan metabolism, and promotes anxiety in female rodents (Baldwin et al., 2017b; Rock et al., 2018). Although this may appear tangential to the present study objectives, 5-HT also impacts aspects of bone physiology. Bone is sensitive to most neurotransmitters, including 5-HT, which promotes bone accrual when produced by the brain and inhibits bone formation when produced peripherally by the gut (Westbroek et al., 2001; Yadav et al., 2008; Ducy and Karsenty, 2010; Pawlak et al., 2019). LRP5, a co-receptor for Wnt signaling, also controls 5-HT synthesis in the gut, and the inhibition of *lrp5* decreases bone mass in mice (Yadav et al., 2008). 5-HT also controls the transcription factor FOXO1 which forms separate complexes with either ATF4, to promote osteoblast proliferation, or CREB, to inhibit proliferation (Rached et al., 2010; Pawlak et al., 2019). The ATF4 complex is favored in environments of increased 5-HT (Rached et al., 2010; Pawlak et al., 2019). Similarly, the non-collagenous protein hormone OCN is produced and secreted exclusively by osteoblasts and primarily acts locally to influence matrix mineralization in skeletal tissue and can influence whole-system metabolism (Guntur and Rosen, 2012; Agas et al., 2013; Oldknow et al., 2015; Wei and Karsenty, 2015; Shan et al., 2019). The main osteocalcin target receptor, Gpr158, is expressed in the hypothalamus and pituitary gland, where it acts to promote spatial learning and memory and inhibits anxiety-like behavior.

Because, in its own internal studies, the chemical manufacturer found adverse skeletal effects in rats exposed to only the BFR components, albeit at doses at much higher exposures, we hypothesized that the BFRs could be most consequential (MPI Research, 2008). At doses of 100 mg/kg/day, inappropriate fusion of cervical vertebral neural arches was observed, and at doses of 300 mg/kg/day, further ossification variations in cervical vertebral neural arches, incomplete ossification of the skull, and unossified sternum were observed (MPI Research, 2008). We also hypothesized that the BFRs and OPFRs might induce unique sex- and FR class-specific effects on bone morphology and circulating serum markers, given their propensity to be metabolically disruptive *in vitro* and in zebrafish assays.

## Materials and methods

### Animal care and maintenance

All tissues were collected from animals used in a prior, published study devised to assess brain and behavior-related outcomes (Witchey et al., 2020). The full methods are detailed in the prior study, but, briefly, Wistar rats were obtained from Charles River (Raleigh, NC) and bred in-house in humidity- (40%–60%) and temperature- (25°C) controlled rooms under a 12:12 light:dark cycle in the AAALAC-approved Biological Resource Facility at North Carolina State University. The animals were maintained in accordance with the best practices to minimize exposure to unintended EDCs which include the use of glass water bottles with metal sippers, a phytoestrogen-free diet (Teklad 2020, Envigo), woodchip bedding, and polysulfone caging. The experiment was designed and described using the Animal Research: Reporting of *In Vivo* Experiments (ARRIVE) Guidelines Essential 10 Checklist for Reporting Animal Research to ensure the highest standard of reporting for animal-based research (Kilkenny et al., 2010).

### Dosing

Dams were orally dosed using concentrated chemical solutions prepared in Dr. Heather Stapleton’s laboratory at Duke University. Dose selection was based on the previous work establishing transplacental and lactational transfer of FM 550 chemicals, and neurobehavioral effects were observed (Phillips et al., 2016). Our prior work established behavioral and sex-specific skeletal effects in rats developmentally exposed to 1,000 µg FM 550 via the dam (Macari et al., 2020). Here, we doubled that dose to obtain information at a different dose and thus contribute to the generation of dose–response information for multiple endpoints. FM 550 is approximately 50% OPFR and BFR chemicals, so to proportionally represent the levels of each in the full mixture, the OPFR and BFR groups were dosed at 1,000 µg. Thus, the four experimental groups were sesame oil vehicle, 2,000 µg FM 550 (∼6.6 mg/kg bw/day), OPFR 1,000 µg/day (∼3.3 mg/kg bw/day), and BFR 1,000 µg/day (∼3.3 mg/kg bw/day). The doses were absolute and not by body weight of individual dams.

The dams were orally exposed daily beginning at 72 h after mate pairing, throughout gestation, and until PND 21 (day of offspring weaning). Control, OPFR, and BFR solutions were dispensed in 20 µL volumes, and FM 550 solution was dispensed in a 40 µL volume on approximately 1/8 of a wafer cookie, which the dams readily consumed. The chemical composition of OPFR, BFR, and FM 550 dosing solutions is presented in Table 1. Dosing was not performed blinded because the FM 550 group had a volume difference, and we preferred to dose the control animals first to minimize the risk of cross-contamination. At PND 21, offspring were weaned into two same-sex littermate groups and housed under the same environmental conditions as their parents (Witchey et al., 2020). The study animals were humanely euthanized by carbon dioxide asphyxiation and rapid decapitation at PND 250 (8 months of age) in agreement with the NCSU Institutional Animal Care and Use Committee (IACUC) animal welfare protocol. The offspring were randomly selected from a total of 10 control, eight OPFR, eight BFR, and nine FM 550 dams with the goal of using only one animal per sex per litter whenever possible. The final exposure group sizes were as follows: control (N = 16; 8 M and 8 F), OPFR (N = 16; 8 M and 8 F), BFR (N = 16; 8 M and 8 F), and FM 550 (N = 18; 8 M and 10 F). All animals, except two control males, two control females, two BFR males, two OPFR males, one OPFR female, one FM 550 male, and one FM 550 female, were siblings. Because our prior study showed significant effects on male skeletal composition, two control males and two FM 550-exposed males from the initial study were included in the assessment described herein to validate our modified approach using the newer technology. Animal assignments, including the four animals included from the pilot study, are listed in Table 2.

**TABLE 1:** Individual chemicals present in the OPFR, BFR, and FM 550 dosing solutions. The estimated mass fraction is adapted from Phillips, et al., 2017.

		Chemical	CAS number	Estimated mass fraction (*w/w*) in FM 550 (%)
Firemaster 550	OPFRs	Triphenyl phosphate (TPHP)	26040-51-7	19.8
		2-Isopropylphenyl diphenyl phosphate (2IPPDPP)	28108-99-8; 93925-53-2; 64532-94-1	11.8
		3-Isopropylphenyl diphenyl phosphate (3IPPDPP)	69515-46-4	1.7
		4-Isopropylphenyl diphenyl phosphate (4IPPDPP)	55864-04-5	2.3
		2,4-8 Diisopropylphenyl diphenyl phosphate (24DIPPDPP)	----	11.0
		Bis(2-isopropylphenyl) phenyl phosphate (B2IPPPP)	69500-29-4	5.1
		Bis(3-isopropylphenyl) phenyl phosphate (B3IPPPP)	69500-30-7	2.1
		Bis(4-isopropylphenyl) phenyl phosphate (B4IPPPP)	55864-07-8	0.3
		Tris(3-isopropylphenyl) phosphate (T3IPPP)	72668-27-0	0.3
	BFRs	2-Ethylhexyl-2,3,4,5-tetrabromobenzoate (EH-TBB)	183658-27-7	29.7
		Bis(2-ethylhexyl)-2,3,4,5-tetrabromophthalate (BEHTEBPA)	26040-51-7	13.9

**TABLE 2:** Tissue sources. Experimental animals were derived from a prior, published study referred to as the “parent study.” Additionally, we included four animals from a published skeletal study to validate the CT methodology.

	Skeletal study Macari, S. et al. (2020)	Parent study Witchey, S.K. et al. (2020)
Total sample size (dams): pups	Ctrl: (8): 4 M and 4 F BFR: --- OPFR: --- FM 550: (7): 4 M and 4 F	Ctrl: (10): 8 M and 8 F
		BFR: (8): 8 M and 8 F
		OPFR: (8): 8 M and 8 F
		FM 550: (9): 8 M and 10 F
Pups used herein for the CT scans	Ctrl: 2 M and 0 F BFR: --- OPFR: --- FM 550: 2 M and 0 F	Ctrl: 8 M and 8 F
		BFR: 8 M and 8 F
		OPFR: 7 M and 7 F
		FM 550: 8 M and 10 F
Pups used herein for the 5HT and OCN assessments	---	Ctrl: 8 M and 8 F
		BFR: 8 M and 8 F
		OPFR: 7 M and 7 F
		FM: 8 M and 10 F
Data for serum ALP and calcium reprinted from the prior study	---	Ctrl: 8 M and 8 F
		BFR: 8 M and 8 F
		OPFR: 7 M and 7 F
		FM: 8 M and 10 F

### X-ray micro-computed tomography imaging

Either the left or right leg of the animal was surgically removed at the hip joint, gently cleaned of skin and auxiliary soft tissue, and submerged in 10% neutral-buffered formalin (NBF). Specimens were randomly assigned a unique ID to blind the user responsible for scanning and analyzing the specimens. The specimens were imaged in 10% NBF in 50-mL conical vials gently immobilized to prevent movement of the specimen during the scan. CT imaging of either the left or right femur was performed using a high-resolution Xradia 510 Versa X-ray microscope (Zeiss, Germany). The imaging conditions were designed in agreement with the guidelines for CT imaging of biological specimens (Bouxsein et al., 2010). Scanning was conducted in 360° rotation with a binning value of 1, LE3 filter, pixel size of 15.35 µM, ×0.4 objective, exposure time of 10 s, and 60 kv/83 uA source to collect 1,600 individual projections per scan.

### CT analysis

The scans were analyzed using Dragonfly Pro software (Object Research Systems, Canada). All scans and analyses were conducted by a blinded user. The femur and a subregion in the distal femur were selected as regions of interest. The femur was digitally isolated to avoid the tibia, fibula, and patella which were included in the complete scan of the leg but not the analysis. In concordance with our previous study, which focused on the distal femur, a z-stack of 100 images immediately below the growth plate was used to digitally create a subregion of interest in the distal femur. Cortical, trabecular, and total bone measurements were then conducted in the distal and total femur. The cortical parameters included average cortical area, average cortical fraction, average cortical thickness, endocortical perimeter, and endocortical surface. The trabecular parameters included average trabecular separation, average trabecular thickness, and average trabecular number. The total bone measurements included bone volume, volume fraction, mineral density, average marrow area, total volume, and average total area. These skeletal parameters are described briefly in Supplementary Table S1. Once data were collected for each skeletal parameter, the user was unblinded to exposure and sex in order to complete the statistical analysis.

### Serum metabolite quantification

Serum was obtained from the trunk blood at the time of euthanasia by rapid centrifugation and stored at −80°C until use. Most of the serum was used for the prior study (Witchey et al., 2020); thus, the selected assays leveraged what remained. The serum levels of 5-HT (Enzo Life Sciences, Catalog #ADI-900-175) and osteocalcin (Novus Biologicals, Catalog #NBP2-68153) were quantified using enzyme-linked immunosorbent assays (ELISAs) according to the manufacturer’s instructions. The serotonin assay used the serum diluted at 1:20, and the osteocalcin assay used the undiluted serum. The plate layout was randomized to minimize inter-assay variation. Additionally, our parent 2020 study conducted a comprehensive analysis of multiple serum markers, including alkaline phosphatase and serum calcium (Witchey et al., 2020), and we report those values herein for the animals examined. Osteocalcin and alkaline phosphatase are established markers of bone health used in clinical and non-clinical settings (Smith et al., 2017). Osteocalcin is produced exclusively by osteoblasts and is frequently used to infer the overall bone health. Alkaline phosphatase (ALP) is an early marker for bone turnover and is critical for the mineralization of bone tissue. A bone-specific isoform of ALP (BAP) is the most direct measurement to reflect ALP in skeletal tissue; however, the total ALP is a commonly accepted proxy to reflect bone health (Smith et al., 2017). Quantitative ELISA for BAP was not available at the time of this study, so we include our previously published ALP data. Similarly, serum calcium can reflect metabolism within skeletal tissue itself.

### Statistical analysis

The animals used here are a subset from a larger parent study [Table 2; (Witchey et al., 2020)]. The weight, ALP, and serum calcium data reported herein are only for the subset of animals used for bone structure assessment and not for the full animal set in the parent study.

The data are presented as the mean ± standard error of the mean (SEM). Statistical analysis was performed using GraphPad Prism, version 9.3.1 (San Diego, CA, United States). Male and female datasets were analyzed independently. A Shapiro–Wilk test was conducted to determine a normal distribution of data. Grubbs’ outlier test (*α* = 0.05) was performed for all endpoints where possible, and outliers were removed on a case-by-case basis if they were found to disrupt normal distribution. To preserve transparency of data reporting, the outliers were kept in the dataset if they did not affect significance or normal distribution. One male and one female from the OPFR exposure group were entirely excluded due to the total body weight exceeding two standard deviations from the mean, which reduced the final animal numbers to 7 M and 7 F within the OPFR exposure group.

For each measurement, the controls were compared using Student’s *t*-test to determine if a sex difference was present. Because significant effects of sex were found for most skeletal parameters, all bone measurements were analyzed by one-way ANOVA within sex and followed by Dunnett’s *post hoc* test. For the data which were not normally distributed, a Kruskal–Wallis test and Dunn’s *post hoc* test were conducted. Non-parametric data were also analyzed through one-way ANOVA and Dunnett’s *post hoc* test. For outcomes where the results are statistically equivalent between both analysis approaches, the results of Dunnett’s test are referred to in the text for consistency in data reporting. Significance was established at *p* ≤ 0.05. The effect size was calculated using Cohen’s d and classified as small (0.2), medium, (0.5), or large (0.8).

## Results

### Body weight

As expected and consistent with our previous results (Macari et al., 2020), the control males were heavier than the control females at the time of euthanasia (t = 8.097, df = 14, *p* ≤ 0.001); however, no statistically significant effect of exposure on body weight was found for either sex [males: (F _(3, 27)_ = 2.34, *p* = 0.09); females: (F _(3, 29)_ = 1.358, *p* = 0.27), Table 3].

**TABLE 3:** Average total body weight (in grams) of experimental animals at the time of euthanasia. Males were larger than females, as expected, and no effect of exposure on body weight was found for either sex.

	Control	BFR	OPFR	FM 550
Male	748.8 ± 25.7 g (*n* = 8)	695.9 ± 22.3 g (*n* = 8)	692.8 ± 9.8 g (*n* = 7)	755.6 ± 23.5 g (*n* = 8)
Female	468.7 ± 23.1 g*** (*n* = 8)	460.6 ± 24.4 g (*n* = 8)	412.0 ± 12.8 (*n* = 7)	413.9 ± 18.1 g (*n* = 10)

***p ≤ 0.001 between opposite sex conspecifics.

### Validation of the CT approach

**FIGURE 1:**
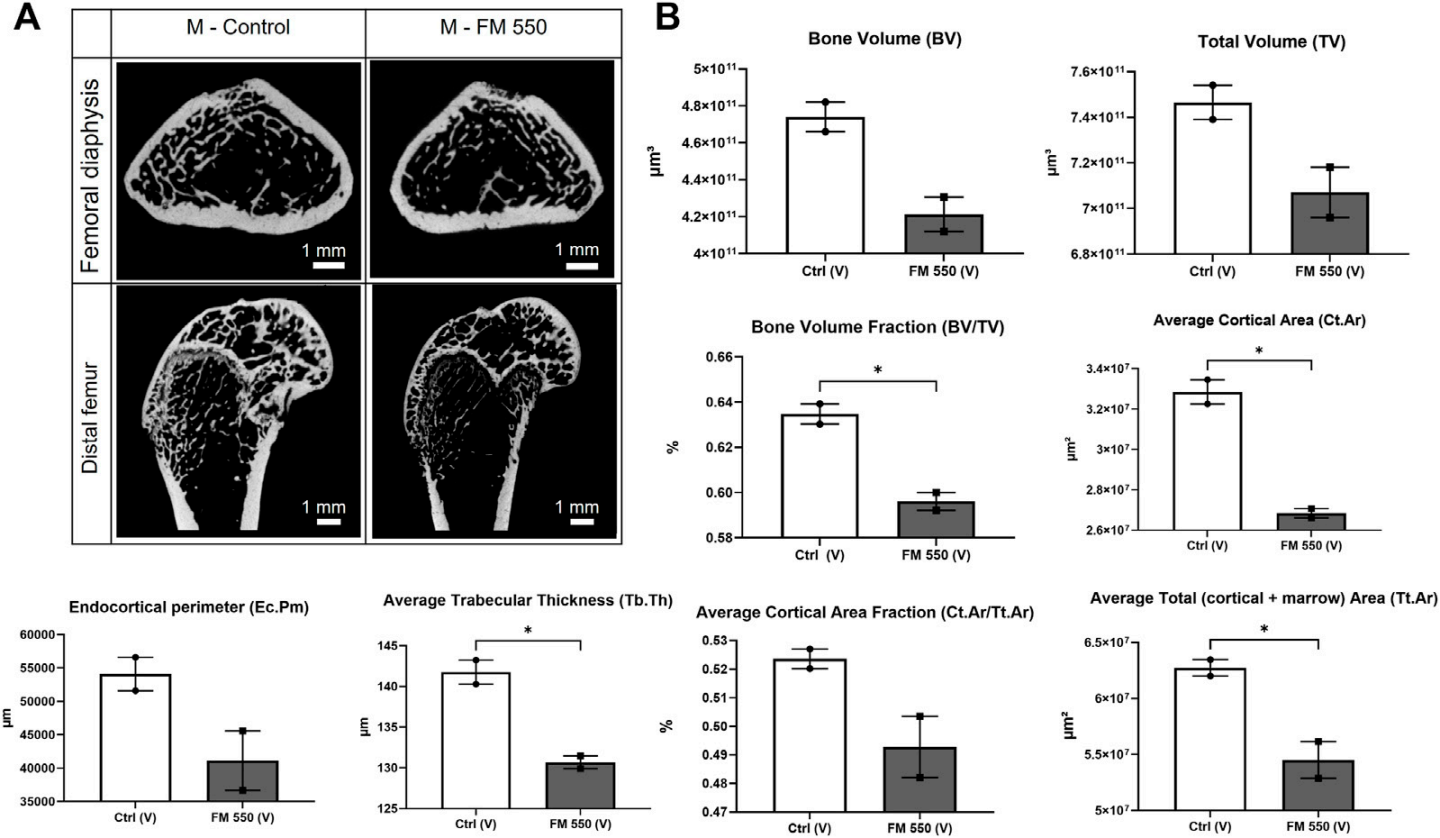
**(A)** Cross sections of the femoral diaphysis and distal femur representative of the sampled area of a control male and a FM 550-exposed male from our 2020 skeletal study scanned using the newer technology employed in this study. This approach was taken to validate the new CT methods. **(B)** Replicating our 2020 findings using different instrumentations, As was found in the original study, FM 550-exposed males had significantly less bone volume fraction, cortical area, total area, and thinner trabecular bone than control males. Bone volume, total volume, endocortical perimeter, and average cortical area fraction showed similar trends but were not statistically significant, given the extremely small sample size (*n* = 2 per group). Bar graphs represent the mean ± SEM; individual animals are represented by icon points. Data were analyzed using Student’s *t*-test. *p ≤ 0.05.

Because we have previously shown skeletal composition of the distal femur in adult males to be significantly affected by developmental exposure to FM 550, two control males and two FM 550 males from the 2020 study were included to validate our approach 1) in the full femur, along with the distal femur, and 2) using newer CT technology (Baldwin et al., 2017b; Macari et al., 2020). The full and distal femurs of these four males were scanned and analyzed as described herein, and the results were compared via Student’s *t*-test. The representative CT images of this subset are included in Figure 1. In agreement with the 2020 study, we again found that the FM 550-exposed males from that study had decreased bone volume fraction (t = 6.48; *p* ≤ 0.02), decreased average cortical area (t = 9.41; *p* ≤ 0.01), decreased trabecular thickness (t = 6.61; *p* ≤ 0.02), and decreased average total area (t = 4.58; *p* ≤ 0.04) in the total femur (Macari et al., 2020). While not every skeletal parameter assessed replicated the findings in our pilot assessment (likely due to the very small sample size used for this validation exercise), we successfully and consistently confirmed an abnormal skeletal phenotype in FM 550-exposed males within the full femur using the newer technology and modified approach.

### Bone structure assessment

For the assessment of the total femur, one BFR male was removed from the measurements of bone volume, endocortical surface, periosteal surface, and total volume, and one control male was removed from average trabecular separation measurement. One OPFR-exposed female was removed from measurements of average cortical thickness, periosteal surface, and total volume. One control female and one BFR-exposed female were removed from the measurement of BMD for being statistical outliers and influencing normal distribution.

**FIGURE 2:**
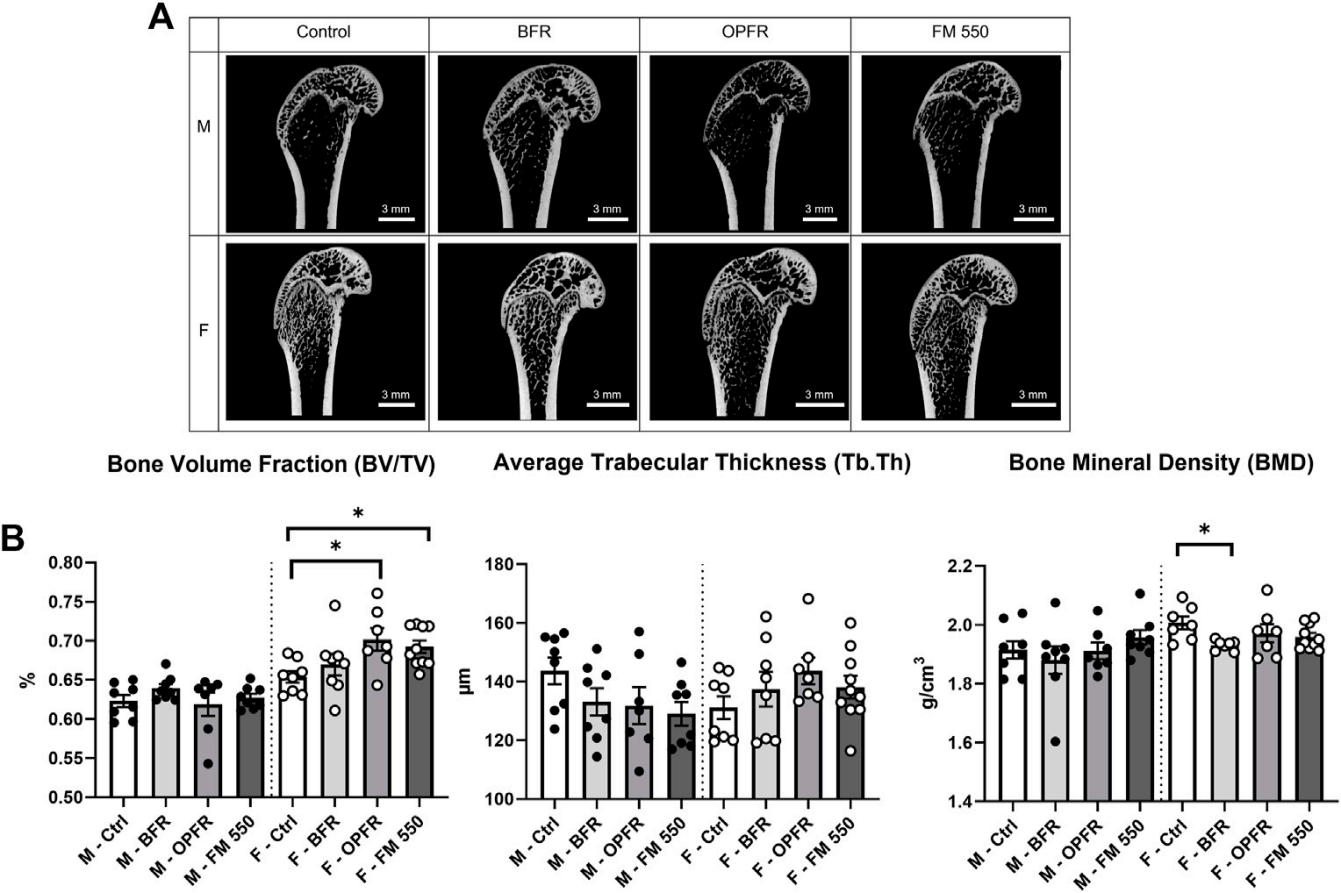
**(A)** Sagittal cross sections of the distal area of the full femur and representing the sampled area, showing the gross structure, and highlighting trabecular and cortical bone across sex and exposure groups. **(B)** OPFR- and FM 550-exposed females had greater bone volume fraction than sex-specific controls. Although not statistically significant, FM 550-exposed males had thinner trabecular bone than control males. Bone mineral density in females was more strongly affected than that in males, with the BFR-exposed females having significantly decreased BMD compared to controls. Measurements were recorded using the full femur dataset. Data were analyzed by one-way ANOVA within sex and Dunnett’s *post hoc* test (*n* = minimum 8 per group). Bar graphs represent the mean ± SEM; individual animals are represented by icon points. *p ≤ 0.05.

**FIGURE 3:**
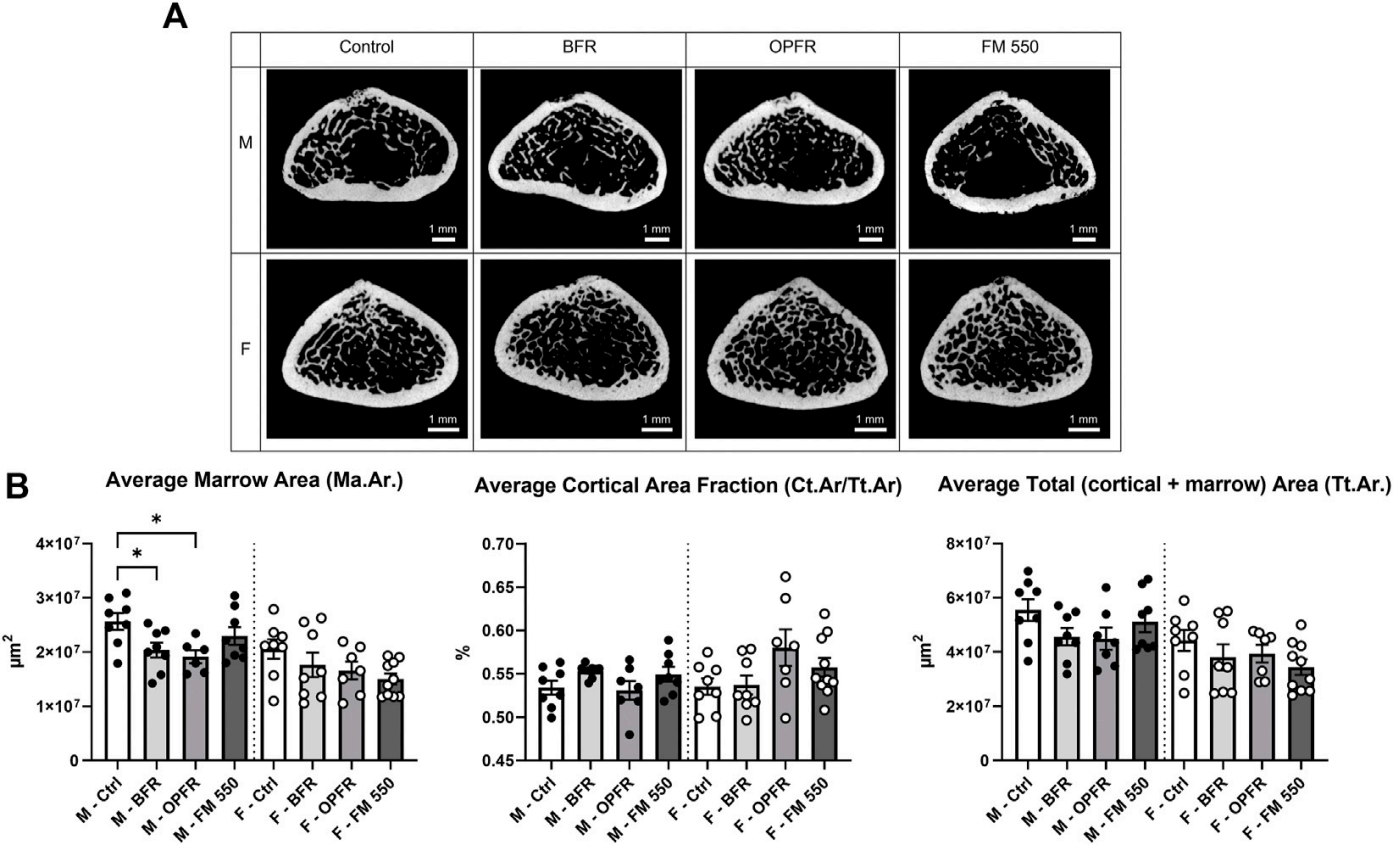
**(A)** Cross sections of the femoral diaphysis, representative of the sampled area and showing the gross structure, highlighting trabecular and cortical bone across sex and exposure groups. **(B)** BFR- and OPFR-exposed males had significantly less marrow area, and, while all exposed females showed similar trends, the effects were not statistically significant. Cortical area fraction and average total fraction were not affected. Measurements were recorded using the full femur dataset. Data were analyzed by one-way ANOVA within sex and Dunnett’s *post hoc* test (*n* = minimum 8 per group). Bar graphs represent the mean ± SEM; individual animals are represented by icon points. *p ≤ 0.05.

Within females, OPFR- (*p* = 0.01, d = 1.57) and FM 550-exposed (*p* = 0.03, d = 1.66) animals had a statistically significant increased bone volume fraction (BV/TV) [F_(3, 29)_ = 3.82, *p* = 0.02, Figure 2]. Although not statistically significant, OPFR-exposed females showed an increased cortical area fraction (Ct.Ar/Tt.Ar) [F_(3, 29)_ = 2.28, *p* = 0.09, d = 1.04, Figure 3], and FM 550-exposed females had decreased marrow area (Ma.Ar) [F_(3, 29)_ = 2.00, *p* = 0.13, d = 1.33, Figure 3]. BFR (*p* = 0.03, d = 1.76, Figure 2) females had significantly decreased bone mineral density compared to the controls [F_(3, 27)_ = 2.38, *p* = 0.09, Figure 2]. In males, BFR-treated animals had decreased marrow area (Ma.Ar) [F_(3, 27)_ = 1.92, *p* = 0.14, d = 1.27, Figure 3], increased total area (Tt.Ar) [F_(3, 27)_ = 1.71, *p* = 0.18, d = 0.95, Figure 3], and decreased bone volume fraction [F_(3, 27)_ = 1.00, *p* = 0.40, d = 0.84, Figure 2], while FM 550-treated males had decreased trabecular thickness (Tb.Th) [F_(3, 27)_ = 1.78, *p* = 0.17, d = 1.19, Figure 2]. A full list of the outcomes measured in the full femur is given in Table 4.

**TABLE 4:** Summary of full femur skeletal outcomes.

	Control			BFR			OPFR			FM 550		
	N	*p*	d	n	*p*	d	n	*p*	d	n	*p*	d
Total volume												
Male	8	**—**	**—**	7	0.27	0.71	7	0.39	0.71	8	0.56	0.65
Female	8	**—**	**—**	8	0.99	0.02	6	0.80	0.36	10	0.99	0.03
Bone volume												
Male	8	**—**	**—**	7	0.63	0.43	7	0.37	0.70	8	0.72	0.49
Female	8	**—**	**—**	8	0.77	0.39	6	0.66	0.64	10	0.16	*0.95*
Bone volume fraction												
Male	8	**—**	**—**	7	0.42	0.84	7	0.97	0.13	8	0.97	0.23
Female	8	**—**	**—**	8	0.61	0.51	7	0.01*	*1.57*	10	0.03*	*1.66*
Average trabecular separation												
Male	7	**—**	**—**	8	0.17	*1.68*	7	0.39	*1.18*	8	0.99	0.04
Female	8	**—**	**—**	8	0.99	0.01	7	0.99	0.19	10	0.79	0.45
Average trabecular thickness												
Male	8	**—**	**—**	8	0.30	*0.81*	7	0.23	*0.80*	8	0.09	*1.20*
Female	8	**—**	**—**	8	0.35	0.45	7	0.14	*1.09*	10	0.28	0.59
Average cortical thickness												
Male	8	**—**	**—**	8	0.92	0.20	7	0.85	0.40	8	0.99	0.16
Female	8	**—**	**—**	8	0.99	0.05	6	0.60	*0.85*	10	0.50	0.53
Average cortical area												
Male	8	**—**	**—**	8	0.33	0.73	7	0.16	*0.94*	8	0.91	0.24
Female	8	**—**	**—**	8	0.59	0.49	7	0.98	0.15	10	0.34	0.71
Average marrow area												
Male	8	**—**	**—**	8	0.08	*1.28*	7	0.18	*0.88*	8	0.54	0.60
Female	8	**—**	**—**	8	0.50	0.50	7	0.28	*0.83*	10	0.06	*1.33*
Average total area												
Male	8	**—**	**—**	8	0.18	*0.95*	7	0.16	*0.94*	8	0.75	0.39
Female	8	**—**	**—**	8	0.51	0.50	7	0.71	0.48	10	0.15	*0.99*
Average cortical area fraction												
Male	8	**—**	**—**	8	0.21	*1.12*	7	0.98	0.12	8	0.36	0.67
Female	8	**—**	**—**	8	0.99	0.07	7	0.07	*1.05*	10	0.47	0.70
Periosteal surface												
Male	8	**—**	**—**	7	0.41	0.58	7	0.54	0.58	8	0.73	0.47
Female	8	**—**	**—**	8	0.99	0.05	6	0.71	0.41	10	0.98	0.14
Endocortical surface												
Male	8	**—**	**—**	7	0.95	0.23	7	0.55	0.51	8	0.99	0.08
Female	8	**—**	**—**	8	0.99	0.01	7	0.99	0.10	10	0.99	0.02
Periosteal perimeter												
Male	8	**—**	**—**	8	0.47	0.64	7	0.35	0.71	8	0.96	0.19
Female	8	**—**	**—**	8	0.54	0.50	7	0.71	0.49	10	0.15	*1.01*
Endocortical perimeter												
Male	8	**—**	**—**	8	0.13	0.42	7	0.13	0.25	8	0.66	0.94
Female	8	**—**	**—**	8	0.65	0.42	7	0.68	0.49	10	0.22	*0.85*
Bone mineral density												
Male	8	**—**	**—**	8	0.99	0.12	7	0.58	0.05	8	0.82	0.54
Female	7	**—**	**—**	7	0.17	*1.76*	7	0.96	0.49	10	0.78	*0.96*

“n” denotes the sample size. Data were analyzed within sex using one-way ANOVA and Dunnett’s *post hoc* test. Significant effects (*p ≤ 0.05) are shown in bold. The effect size was calculated using Cohen’s d, and large effect sizes (d > 0.08) are shown in italics.

In the distal femur, only BFR-exposed males had decreased bone volume fraction [F _(3, 26)_ = 3.715, *p* = 0.02, d = 1.55]. No significant effects were found for females. A full list of the outcomes in the distal femur is provided in Table 5. One exposed male was removed from the measurements of bone volume, average cortical area, average marrow area, endocortical perimeter, endocortical surface, periosteal perimeter, periosteal surface, total volume, and total area. The same animal from the BFR group (one male) was used for all measurements where it was found to be an outlier, which influenced normal distribution. One control male and one control female were removed from the measurement of bone volume fraction for the same reason.

**TABLE 5:** Summary of distal femur skeletal measurements.

	Control			BFR			OPFR			FM 550		
	n	*p*	d	n	*p*	d	n	*p*	d	n	*p*	d
Total volume												
Male	8	**—**	**—**	7	0.72	0.38	7	0.72	0.42	8	0.92	0.26
Female	8	**—**	**—**	8	0.89	0.27	7	0.41	*0.89*	10	0.99	0.06
Bone volume												
Male	8	**—**	**—**	7	0.77	0.38	7	0.69	0.61	8	0.38	0.74
Female	8	**—**	**—**	8	0.68	0.45	7	0.90	0.36	10	0.30	*0.81*
Bone volume fraction												
Male	7	**—**	**—**	8	0.01*	*1.55*	7	0.06	*1.21*	8	0.60	0.68
Female	7	**—**	**—**	8	0.60	0.48	7	0.11	*2.03*	10	0.11	*1.34*
Average trabecular separation												
Male	8	**—**	**—**	8	0.28	0.79	7	0.76	0.37	8	0.76	0.14
Female	8	**—**	**—**	8	0.87	0.24	7	0.31	0.75	10	0.06	*1.24*
Average trabecular thickness												
Male	8	**—**	**—**	8	0.99	0.09	7	0.93	0.29	8	0.50	0.65
Female	8	**—**	**—**	8	0.84	0.41	7	0.36	*0.80*	10	0.51	0.53
Average cortical thickness												
Male	8	**—**	**—**	8	0.59	0.20	7	0.99	0.06	8	0.84	0.28
Female	8	**—**	**—**	8	0.75	0.39	7	0.96	0.24	10	0.66	0.45
Average cortical area												
Male	8	**—**	**—**	7	0.76	0.36	7	0.82	0.39	8	0.43	0.64
Female	8	**—**	**—**	8	0.98	0.11	7	0.70	0.56	10	0.83	0.32
Average marrow area												
Male	8	**—**	**—**	7	0.54	0.58	7	0.53	0.53	8	0.83	0.34
Female	8	**—**	**—**	8	0.88	0.30	7	0.86	0.40	10	0.92	0.25
Average total area												
Male	8	**—**	**—**	7	0.18	0.38	7	0.08	0.39	8	0.30	0.59
Female	8	**—**	**—**	8	0.93	0.22	7	0.41	*0.89*	10	0.99	0.05
Average cortical area fraction												
Male	8	**—**	**—**	8	0.95	0.19	7	0.82	0.32	8	0.99	0.02
Female	8	**—**	**—**	8	0.93	0.23	7	0.99	0.02	10	0.86	0.29
Periosteal surface												
Male	8	**—**	**—**	7	0.51	0.53	7	0.46	0.67	8	0.61	0.57
Female	8	**—**	**—**	8	0.79	0.36	8	0.34	*1.13*	10	0.99	0.06
Endocortical surface												
Male	8	**—**	**—**	7	0.82	0.36	7	0.95	0.20	8	0.93	0.26
Female	8	**—**	**—**	8	0.52	0.54	8	0.83	0.50	10	0.86	0.33
Periosteal perimeter												
Male	8	**—**	**—**	7	0.27	0.51	7	0.13	0.61	8	0.39	0.69
Female	8	**—**	**—**	7	0.98	0.12	7	0.43	*0.84*	10	0.99	0.02
Endocortical perimeter												
Male	8	**—**	**—**	7	0.68	0.47	7	0.89	0.26	8	0.99	0.15
Female	8	**—**	**—**	8	0.73	0.38	8	0.87	0.38	10	0.90	0.29

“n” denotes sample size. Data were analyzed within sex using one-way ANOVA and Dunnett’s *post hoc* test. Significant effects (*p ≤ 0.05) are shown in bold. The effect size was calculated using Cohen’s d, and large effect sizes (d > 0.08) 0.8 are shown in italics.

### Serum markers

#### OCN

**FIGURE 4:**
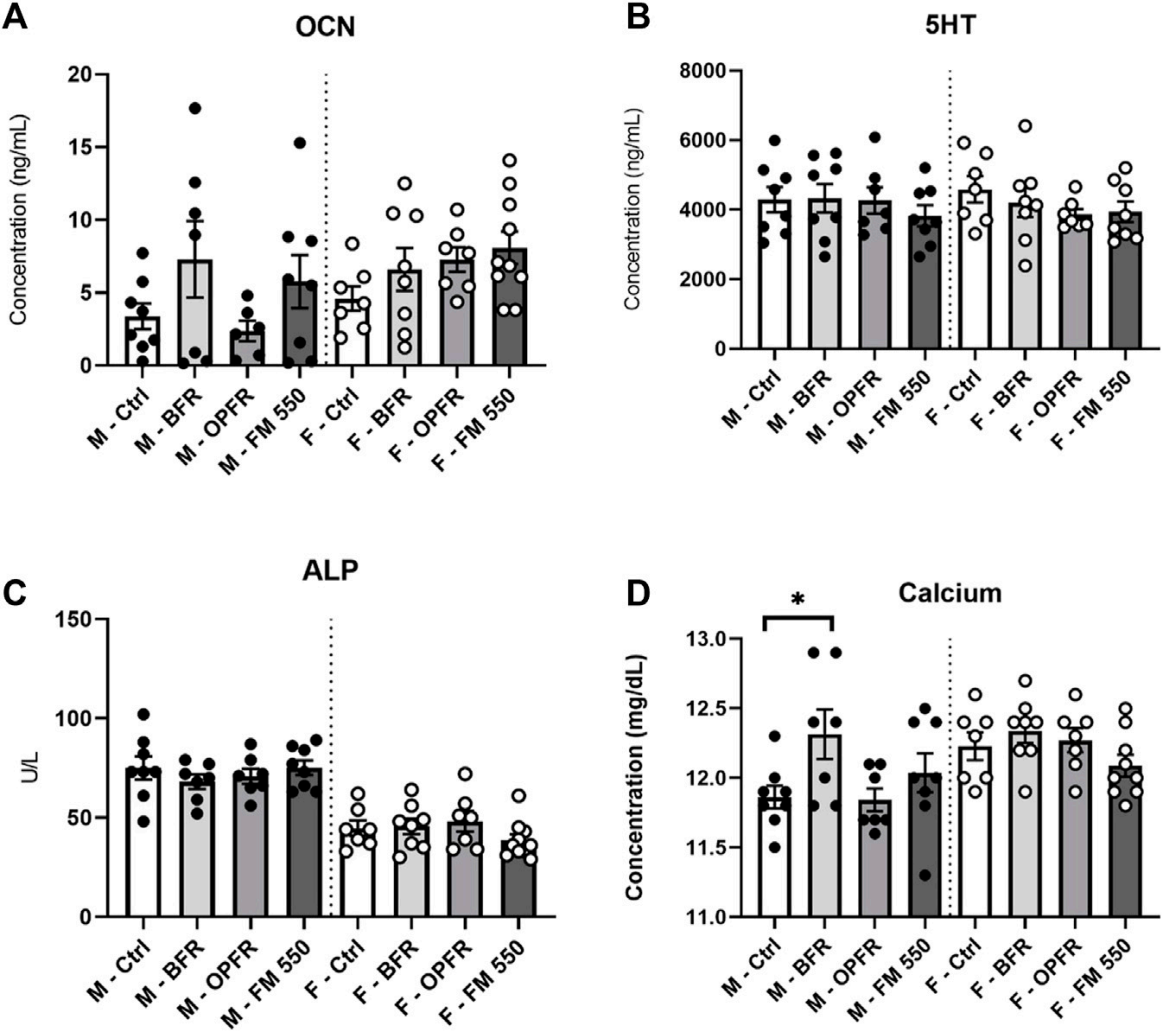
Serum was analyzed via VetScan or ELISA analysis. Expected sex differences were found for ALP and calcium, and BFR-exposed males showed increased calcium levels. No sex- or exposure-related effects were found for serotonin. No sex difference was present for osteocalcin, and no exposure effects were identified. Bar graphs represent the mean ± SEM; individual animals are represented by icon points. Data were analyzed by one-way ANOVA within sex and Dunnett’s *post hoc* test (*n* = minimum 8 per group). *p ≤ 0.05.

No sex differences were found for the average OCN levels in controls, where control males showed 3.37 ng/mL and control females showed 4.59 ng/mL (t = 0.99; df = 13). Although not statistically significant, OCN values for FM 550-exposed females were greater than those for controls at 8.56 ng/mL [F_(3, 28)_ = 2.00, *p* = 0.13, d = 1.34, Figure 4]. The average levels were also elevated in the BFR and OPFR groups at 6.59 ng/mL and 6.67 ng/mL, with effect sizes of 0.58 and 0.90, respectively, although the values were not statistically significant. A technical error occurred with one FM 550-exposed female and could not be analyzed. No difference was found for exposed males, although qualitatively, the OPFR males appeared to have a bimodal distribution [F_(3, 27)_ = 0.8102, *p* = 0.49, Figure 4]. The average OCN values of BFR-, OPFR-, and FM-exposed males were 7.29 ng/mL, 4.60 ng/mL, and 5.77 ng/mL with effect sizes of 0.77, 0.30, and 0.59, respectively.

#### Serotonin

5-HT could not be measured for one control female due to a technical error. No sex difference was found between controls (t = 0.56; df = 13) nor were significant effects of exposure found for males [F_(3, 27)_ = 0.53, *p* = 0.66] or females [F_(3, 29)_ = 0.33; *p* = 0.80, Figure 4].

#### Alkaline phosphatase

ALP could not be measured for one control female due to a technical error. As reported previously and restated here, serum ALP levels were higher in control males than those in females (t = 4.22, df = 13, *p* = 0.001), and no exposure-related effects were found in either males [F_(3, 26)_ = 0.36, *p* = 0.78] or females [F_(3, 29)_ = 1.31, *p* = 0.28, Figure 4]. The average values were 75 U/L for control males and 44.71 U/L for control females. In exposed males, BFR-, OPFR-, and FM 550-exposed animals had average values of 70.83 U/L, 70.5 U/L, and 75.13 U/L, along with effect sizes of 0.31, 0.33, and 0.01, respectively. One BFR-exposed male was removed for being a statistical outlier. In exposed females, BFR- and OPFR-exposed females had average values of 45.63 U/L and 48.38 U/L and effect sizes of 0.08 and 0.31, respectively. FM 550-exposed females had a lower average of 38.6 U/L and effect size of 0.63, although the value was not statistically significant.

#### Calcium

Calcium could not be measured for one control female due to a technical error. As reported previously, we restate here that an expected sex difference was found within controls, with females having higher serum calcium than males (t = 2.864, df = 13, *p* = 0.01). Females had 12.23 mg/dL, while males had 11.86 mg/dL. However, no effect of exposure was found for serum calcium in females [F_(3, 29)_ = 0.22, *p* = 0.87, Figure 4]. The value sizes for BFR-, OPFR-, and FM 550-exposed females were 12.34 mg/dL, 12.24 mg/dL, and 12.24 mg/dL, respectively. Small effect sizes were found for females at 0.44, 0.04, and 0.02 for BFR-, OPFR-, and FM 550-exposed animals, respectively. Within males, serum calcium levels were greater in BFR-exposed males than controls at 12.31 mg/dL [F_(3, 27)_ = 2.54, *p* ≤ 0.05, d = 1.242, Figure 4]. OPFR males had slightly lower calcium levels than controls at an average of 11.90 mg/dL (d = 0.16), while FM 550-exposed males had slightly higher calcium levels than controls at 12.04 mg/dL (d = 0.55).

## Discussion

Gestational and lactational exposure to FM 550 or its OPFR or BFR components resulted in sex- and chemical-specific evidence of altered skeletal tissue organization in both the full and distal femur analyses. Unexpectedly, neither the OPFRs nor the BFRs produced a profound phenotype, suggesting that each contributes aspects to the effects of the full mixture. This finding is significant because the OPFR and BFR components are used in a wide variety of applications in different combinations. The mechanisms by which each class contributes to the structural phenotype remain to be established. The FM 550 phenotype in males was similar but not as severe as in our prior study using a lower FM 550 dose and assessing animals as younger adults (Macari et al., 2020). Critically, we show that we were able to recapitulate the phenotype using the revised approach on a subset of the males from the prior study and demonstrate that the discordance is not due to technical issues related to the updated CT approach but, instead, likely results from other experimental differences, including the older age at analysis and the higher FM 550 dose used. A comprehensive study incorporating additional doses and ages at examination to fully characterize the trajectory of the phenotype over the dose and life stage is merited. The present study expands on our prior study by elucidating how OPFR and BFR components of the FM 550 mixture sex-specifically disrupt skeletal development and by contributing hazard information at a higher dose. Our data also add to the limited but growing body of literature focusing on bone as a potential target for endocrine disruption.

Fetal skeletogenesis in the rat begins on GD 15, and the skeleton is fully formed by PND 2 (DeSesso and Scialli, 2018). Rapid bone growth and development occur postnatally with the appearance of the primary ossification center in the femur on PND 8. Thus, our exposure window from GD 0 to PND 21 fully encompassed all sensitive time points for early skeletal development (DeSesso and Scialli, 2018). Importantly, the parent study from which the animals were obtained was designed to focus primarily on behavioral outcomes, many of which rely on healthy motor function. Those data showed no indication of any gross mechanical impairment, and no animals showed any signs of impaired movement or mobility during behavioral tasks conducted on or around PND 90 (Witchey et al., 2020). Additionally, no evidence of impaired gross motor function was observed in any of our other prior behavioral studies on FM 550 (Patisaul et al., 2013b; Baldwin et al., 2017a; Gillera et al., 2022). Regardless, disrupted femoral skeletal organization was apparent in PND 250 FM 550 males in our prior study and less so in the older males tested herein. Collectively, this suggests that structural effects may be more pronounced in younger animals, particularly males, but are not severe enough to influence gross motor activity. *Ex vivo* biomechanical testing would be useful to test this posit across development in future studies, as would the assessment of fine motor activity. Vulnerability to skeletal degeneration at an advanced age is also a possibility that should be examined in future studies.

While sex-specific effects were anticipated, why the effects in females were significant here and not in the prior study is unclear, as is why females appeared to be more greatly affected by the OPFRs, while the males appeared to be more affected by the BFRs. Two main differences between the present and our prior skeletal study are dose and age at testing. One hypothesis regarding the males is that the phenotype previously observed is not produced at the higher dose used here due to yet-to-be described compensatory mechanisms not triggered at lower exposures. Non-monotonic dose responses (NMDRs) commonly occur with EDCs via multiple mechanisms (Vandenberg et al., 2012). In some instances, repair mechanisms are activated at higher, but not lower, more human-relevant doses, and this may be the case here. In nuclear hormone receptor binding assays, FM 550 shows a strong NMDR at PPARy, which is suggested to be driven by the OPFR components in the mixture (Belcher et al., 2014). Alternatively, the animals in this study were approximately 3 months older than the animals assessed in the prior study. Thus, it is possible to resolve the male skeletal phenotype through growth and remodeling.

Why a phenotype emerged in the older females when we did not detect one previously in younger adults is unknown. Estrogen is largely protective of bone health; thus, one hypothesis is that gonadal estrogens may mask exposure-related effects in younger females that recede with age as estradiol (E2) levels naturally decline (Imai et al., 2013). In males, estrogens are also protective. ERα-mediated signaling is critical for skeletal maintenance, with aromatase producing estradiol locally from circulating testosterone (Bouillon et al., 2004; Sinnesael et al., 2011; Imai et al., 2013). Aromatase knockout (ArKO) mice display a strong sex-specific skeletal phenotype, with ArKO females showing higher rates of bone turnover than males (Oz et al., 2000; Oz et al., 2001). The assessment of ER levels in bone was beyond the scope of this study but should be examined in future works.

Other EDC studies report evidence of improvement with age. In a similar study with gestational and lactational bisphenol-A (BPA) exposure, skeletal insults recorded in males at 5 weeks of age were resolved by 52 weeks, suggesting that the phenotype may be reversible or resolved through remodeling as the animal ages (Lind et al., 2017; Lind et al., 2019). Similar studies with bone-damaging pharmaceuticals also show that skeletal recovery following insult is possible. For example, intraperitoneal injection of a chemotherapy agent induced apoptosis and inhibited cartilage proliferation at the growth plate in 6-week-old male rats (Xian et al., 2004; Xian et al., 2006). However, by 10–14 days post-exposure, the proliferative and hypertrophic zones of the growth plate returned to normal thickness, and by 4–10 days post-exposure, expression of bone matrix proteins and growth factors increased (Xian et al., 2006). Future studies will assess animals at earlier stages of development (<PND 100) to establish a timeline for the skeletal phenotype in each sex. The present studies were conducted during the peak of COVID-related restrictions, and associated logistical limitations prevented planned histological assessment of skeletal tissue in this study; hence, the quantification of osteoblast, yellow marrow, and osteoclast populations will be desirable in future studies.

The effects of FR exposure on serum biomarkers were minimal, but the identification of expected sex differences confirms that the study was sufficiently powered to detect biologically meaningful effects. Expected sex differences in serum calcium and ALP levels were found, with males having higher ALP and lower serum calcium levels than females. FM 550-exposed females had higher concentrations of osteocalcin than controls, and BFR-exposed males had increased serum calcium compared to controls. Osteocalcin is produced exclusively from osteoblasts and primarily deposited in the bone matrix. Increased osteocalcin levels in the FM 550 females could suggest increased bone turnover activity but would need to be confirmed (Agas et al., 2013; Hlaing and Compston, 2014). Because increased serum calcium can result from many biological factors, why serum calcium was higher in BFR-exposed males is unclear. Involvement of the thyroid hormone and parathyroid hormone is a possibility, as thyroid hormone disruption is a well-known consequence of BFR exposure (Darnerud, 2008; Wang et al., 2019). Increased OCN in FM 550-exposed females and increased calcium in BFR-exposed males do not correlate with any single skeletal phenotype. However, the relationship between the sex and exposure group is consistent with the previous results for skeletal effects, where BFR exposure more commonly affected males and FM 550 exposure more commonly affected females.

The sex-specific effects of developmental FR exposure found are consistent with known sex differences in skeletal physiology and many other EDCs. In mice exposed to either 200 μg/kg body weight BPA or bisphenol-S (BPS) orally via the dam throughout gestation and lactation, only the BPA-exposed males had an impaired skeletal structure (Dirkes et al., 2021a; Dirkes et al., 2021b). The effects on females were minimal. Similarly, male and female mice exposed through diet to 500 μg/kg diethylstilbestrol (DES) or greater had increased trabecular bone formation in the proximal femur; however, only males showed increased bone formation in the sternum (McAnulty and Skydsgaard, 2005). In a rat model of developmental exposure, dams were exposed to approximately 0.06, 20, and 60 mg/kg body weight/day of a PBDE and hexabromocyclododecane (HBCD) mixture incorporated in chow, throughout gestation and lactation (Berger et al., 2014). Offspring exposed to any of the BFR doses had increased incidents of skeletal variations including incomplete ossification of the sternum or fusion of the vertebral column at GD 20 (Berger et al., 2014). On PND 4, offspring had delayed ossification in phalanges and cervical vertebrae, although neither were sex-specific effects reported nor were later stages of development assessed for persisting skeletal malformations (Tung et al., 2016). All serum biomarkers examined showed no difference in control levels by PND 210, an outcome concordant with our serum data at PND 250 and supporting the hypothesis that the adverse early-life skeletal effects can improve with age (Tung et al., 2016).

To the best of our knowledge, skeletal outcomes of FM 550 exposure have not been assessed in human studies, although a limited but growing number of studies include bone structure assessment in populations exposed to broader categories of EDCs. Globally, BFR and OPFR chemicals are routinely detected in biological tissues such as nail clippings, urine and feces in adults and toddlers, and breastmilk in women (Norén and Meironyté, 2000; Hoffman et al., 2014; Kim et al., 2014; Sahlström et al., 2015). The average human exposure to FM 550 or its OPFR and BFR components varies widely, partially due to consumer and lifestyle choices and occupational exposure, although the two brominated chemicals in the BFR portion of FM 550, EH-TBB and BEH-TBPH, were quantified at geometric mean concentrations of 315.1 and 364.7 ng/g, respectively, in house dust samples of homes in the United States (Stapleton et al., 2008; Hoffman et al., 2014). In a similar study, the geometric mean concentration of TPHP in US house dust extract was quantified at 7,360 ng/g (Stapleton et al., 2009). Although controversial, the ratio between the length of the second and fourth digits is sometimes used as a population-level indicator of prenatal androgen exposure, with a higher digit ratio suggesting feminization (Lutchmaya et al., 2004; Zheng and Cohn, 2011; Auger et al., 2013). A single study found that toddlers prenatally exposed to PBDEs had a higher second digit-to-fourth digit ratio as measured at 4 years of age (Chen et al., 2021b). While digit ratios are not a conclusive marker of skeletal disruption, it is interesting to note that incomplete ossification in the phalanges was observed in young rats following gestational and lactational PBDE exposure (Berger et al., 2014).

The mechanisms by which FM 550 and its component impair skeletal physiology remain to be elucidated. As a mixture, Firemaster 550 has significant agonistic effects on PPARy, which favors adipogenesis at the expense of osteogenesis, an effect largely driven by the OPEs (Belcher et al., 2014). The BFRs, by contrast, may exert their effects via thyroid hormone disruption (Darnerud, 2008; Legler, 2008). RNA sequencing of cultured limb buds from mice exposed to 10 µM tert-butylphenyl diphenyl phosphate for 3 h revealed downregulated transcripts including *gli1* and *runx3* (Yan and Hales, 2020). Following 24 h of exposure, downregulation of transcripts involved in Hedgehog signaling was further amplified, suggesting a dose-responsive effect (Yan and Hales, 2020). Gli1 is a transcription factor belonging to the family of critical Gli proteins in the Hedgehog signaling pathway including endochondral ossification, and runx3 is critical for chondrocyte maturation also involved in this pathway (Yoshida et al., 2004; Day and Yang, 2008; Ohba, 2020). Tracing the lineage of Gli1-positive cells in mice revealed that Gli1 marks osteoprogenitor cells involved in bone development and repair (Shi et al., 2017). As most bone development and repair is mediated by chondrocytes and deposition of a cartilage matrix, the downregulation of *runx3* may suggest that chondrocyte maturation may be susceptible to OPFR disruption (Yoshida et al., 2004).

Finally, it is possible that vascular attributes could be contributing to the observed phenotypes. Vasculature plays a critical role in maintaining skeletal homeostasis and integrity throughout life, and sex differences in skeletal morphology are considered to be at least partially influenced through the vasculature (Ahluwalia et al., 2014; Jani and Rajkumar, 2006; Kaigler et al., 2003; Ramasamy et al., 2016; Owen-Woods and Kusumbe, 2022; Eriksen, 2010; Goring et al., 2019). Endothelial cells (ECs) release angiocrine factors, which include vascular endothelial growth factors (VEGFs) and fibroblast growth factors (FGFs), to promote and regulate bone growth (Hu and Olsen, 2016; Kaigler et al., 2003; Ramasamy et al., 2016; Owen-Woods and Kusumbe, 2022). Limited studies exist on the ability of FRs to impact EC function, although BFRs and OPFRs have been shown to induce apoptosis and genotoxicity in human umbilical vein ECs (Chen et al., 2020; Saquib et al., 2021). Deletion of bone-derived VEGF in osteoblasts obtained from male mice produced immature mineral precursors in culture, whereas it produced mature mineral precursors from female-derived osteoblasts, suggesting a role of VEGF in influencing the skeletal matrix independent of sex hormones in a sex-specific manner (Goring et al., 2019).

## Conclusion

Incorporation of bone structure assessments into a toxicology testing framework remains relatively rare. Thus, a significant risk to human health from developmental FR exposure, and EDC exposure generally, may possibly be undetected. Hence, our study fills a need for data on how early-life chemical exposures influence adult skeletal outcomes. We found that developmental FM 550 exposure via the dam causes lasting but limited skeletal effects in rats, which are both sex- and chemical class-specific. Adult rats (PND 250) showed deficits in adult femur organization including significantly decreased marrow area, increased bone volume fraction in FR-exposed males, and decreased bone mineral density in FR-exposed females. The presence of skeletal effects in late adulthood suggests the disruption of pathways involved in early skeletal organization, but this needs to be examined in younger animals, as well as the potential mechanisms by which these adverse effects occur. The FRs examined have been shown to disrupt estrogen and thyroid hormone signaling, and this is a relevant point to consider since both estrogen and thyroid hormones are critically involved in skeletal development and maintenance. Further examination of other long bone components, especially the vasculature, is also needed to better elucidate the complete phenotype in each sex across the lifespan.

## Data Availability

The original contributions presented in the study are included in the article/Supplementary Material; further inquiries can be directed to the corresponding author.
