# Supplementary material for: Skeletal effects following developmental flame-retardant exposure are specific to sex and chemical class in the adult Wistar rat
Source: Front Toxicol. 2023 Jul 27;5:1216388. doi: 10.3389/ftox.2023.1216388 (PMC10414991; doi:10.3389/ftox.2023.1216388)
Supplement: Supplementary file 1 [file Table1.DOCX]

**Developmental flame retardant exposure imparts skeletal effects which are specific to sex and chemical class in the adult Wistar rat**

Stacy Schkoda^1^, Brian Horman^1^, Shannah Witchey^2^, Anton Jansson^3^, Soraia Macari^4^, and Heather B. Patisaul^1,5^

^1^Department of Biological Sciences, North Carolina State University, Raleigh, NC 27695, USA

^2^National Toxicology Program, National Institute of Environmental Health Sciences, Research Triangle Park, NC

^3^Analytical Instrumentation Facility, North Carolina State University, Raleigh, NC 27695, USA

^4^Department of Restorative Dentistry, Federal University of Minas Gerais, Belo Horizonte, Minas Gerais, Brazil

^5^Center for Human Health and the Environment, North Carolina State University, Raleigh, NC 27695, USA

*Correspondence: [hbpatisa@ncsu.edu](mailto:hbpatisa@ncsu.edu)

**Key Terms**: Endocrine disrupting chemicals, flame retardants, sex difference, osteotoxicology

**Supplemental Table 1.** Description of skeletal parameters measured. Adapted from Bouxin et al 2010 and Object Research Systems.

| **Measured Parameter** | **Abbreviation** | **Unit** | **Description** |
| --- | --- | --- | --- |
| Total volume | TV | µm³ | The volume of the segmented cortical and marrow areas computed from the input bone segmentation. |
| Bone volume | BV | µm³ | The volume of cortical and trabecular bone computed from the input bone segmentations |
| Bone volume fraction | BV/TV | % | The ratio of the bone volume (BV) to the total volume (TV) |
| Average trabecular separation | Tb.Sp | µm | The mean distance between trabeculae, assessed using direct 3D methods |
| Average trabecular thickness | Tb.Th | µm | The mean thickness of trabeculae, assessed using direct 3D methods. |
| Average cortical thickness | Ct.Th | µm | The mean thickness of cortical bone, assessed using direct 3D methods. |
| Average cortical area | Ct.Ar | µm² | The mean cortical bone area, computed as: cortical volume / length of the segmentation bounding box in the Z axis |
| Average marrow area | Ma.Ar | µm² | The mean marrow (medullary) area computed as: trabecular volume / length of the segmentation bounding box in the Z axis |
| Average total area (cortical bone + marrow) | Tt.Ar | µm² | The total cross-sectional area inside the periosteal envelope measured for each transverse section |
| Average cortical area fraction | Ct.Ar/Tt.Ar | % | The ratio of the mean cortical area to the mean total area |
| Periosteal surface (3D) | Ps.S3D | µm² | The total surface area of the periosteum |
| Endocortical surface (3D) | Ec.S3D | µm² | The total surface area of the endocortex |
| Periosteal perimeter | Ps.Pm | µm | The total perimeter of the periosteum |
| Endocortical perimeter | Ec.Pm | µm | The total perimeter of the endocortex |
